# Valorisation of Pre- and Post-Consumer Cotton, Viscose and Bast-Fibre Textile Waste into Sodium Carboxymethyl Cellulose: A Comprehensive Study of Feedstock Type, Synthesis Route and Product Quality

**DOI:** 10.3390/ma19194101

**Published:** 2026-09-25

**Authors:** Marcin Małek, Norbert Lipka, Marcin Szeliga, Monika Tabor

**Affiliations:** 1 Faculty of Civil Engineering and Geodesy, Military University of Technology (Wojskowa Akademia Techniczna), gen. Sylwestra Kaliskiego 2, 00-908 Warsaw, Poland; 2 CMC S.A., Weteranów 12, 03-172 Warsaw, Poland; norbertlipka@wp.pl (N.L.); m.szeliga@cmcsa.pl (M.S.); monika.maleta@gmail.com (M.T.); 3 Department of Microbiology, Institute of Biology, Jan Kochanowski University, 25-406 Kielce, Poland

**Keywords:** carboxymethyl cellulose, textile waste, cellulose valorisation, degree of substitution, etherification, circular economy, post-consumer cotton, viscosity, SEM/EDS, ANOVA

## Abstract

Sodium carboxymethyl cellulose (Na-CMC) is among the most widely used water-soluble cellulose ethers, yet its industrial production still relies largely on virgin cotton linters and wood pulp. In this work, we systematically converted a broad panel of cellulose-bearing textile wastes—reference fabrics, pre-consumer sewing-room clippings and post-consumer garments made of cotton, viscose, flax, jute and their blends—into Na-CMC through heterogeneous alkalisation and etherification in an isopropanol–water medium. Five synthesis routes were compared, including single-step, stepwise-reagent, hydrogen peroxide (H_2_O_2_) bleaching/degrading, cross-linking (dichloroacetic acid) and sodium monochloroacetate (MCA-Na) variants. Fifty-five separate batch syntheses were characterised for degree of substitution (DS), 2% apparent viscosity, active-substance purity, pH and residual moisture. The mean DS was 0.48 ± 0.07 and did not differ significantly among feedstock classes; used garments yielded similar mean substitution levels to reference fabrics in the statistical comparison. Product purity, in contrast, was lower on average for post-consumer material (71.9%) than for reference fabrics (75.9%), with a broader post-consumer distribution. scanning electron microscopy/energy-dispersive X-ray spectroscopy (SEM/EDS) examination of the feedstocks matched this trend, showing frayed, contaminated post-consumer fibres with sulfur signals and locally elevated aluminium on the fibre surface, which were not observed on the smooth pre-consumer cotton. DS showed moderate positive correlations with purity and log-viscosity. Apparent viscosities spanned four orders of magnitude (2.5–23,100 mPa·s), enabling a single, tunable process to deliver both low- and high-viscosity grades. The moderate DS (reagent efficiency ≈ 42%) is consistent with the monochloroacetate charge and reagent losses to hydrolysis rather than with the textile origin of the cellulose, and hydrogen peroxide lowered viscosity to 2.5–30 mPa·s without a significant loss of substitution. The results demonstrate laboratory-scale conversion of mechanically shredded textile waste into technical-grade CMC spanning a broad viscosity range.

## 1. Introduction

Cellulose is the most abundant renewable biopolymer on Earth, with an estimated annual photosynthetic output of the order of 10^11^ tonnes, and it constitutes the structural backbone of the plant cell wall as well as of the principal natural textile fibres [1,2]. Chemically, it is a linear, syndiotactic homopolymer of D-anhydroglucopyranose units (AGU) joined by β-(1→4) glycosidic bonds; extensive intra- and inter-chain hydrogen bonding between the three hydroxyl groups of each AGU gives rise to a partially crystalline, highly cohesive supramolecular architecture that renders native cellulose insoluble in water and in most common solvents [1,3]. Cotton is the purest natural source, containing roughly 90% cellulose, whereas flax, jute and other bast fibres combine cellulose with hemicellulose, lignin and pectins, and regenerated fibres such as viscose are produced from wood- or cotton-derived cellulose by the xanthate or cuprammonium routes [2,4]. The three reactive hydroxyls per AGU make cellulose a versatile platform for chemical derivatisation, of which etherification to carboxymethyl cellulose (CMC) is industrially the most important, alongside methyl-, hydroxyethyl- and hydroxypropyl-cellulose [3,4].

In its sodium-salt form (Na-CMC, commonly termed cellulose gum), the polymer becomes water-soluble because the introduction of anionic carboxymethyl groups disrupts the native hydrogen-bond network and imparts polyelectrolyte character [5]. These polar, chemically reactive groups underpin the exceptionally broad utility of CMC as a thickener, stabiliser, emulsifier, water-retention agent, film former, suspending aid and rheology modifier. Roughly one quarter of global CMC consumption is in food and beverages, where it acts as a thickener, stabiliser and emulsifier, while the remainder is distributed across the pharmaceutical, cosmetic, detergent, paper, textile, ceramic, oil-drilling and mineral-processing industries [3,6,7]. In froth flotation, for example, CMC is a depressant of magnesium-silicate gangue minerals such as talc and chlorite, with an efficiency that depends on its molecular weight [7], and in the paper industry it improves dry strength and surface sizing. The polymer is non-toxic, biodegradable, hypoallergenic and, for several grades, authorised as a food additive (E 466) and widely used as a pharmaceutical excipient, which broadens its appeal in biomedical contexts [3,8,9].

The single most important quality descriptor of CMC is the degree of substitution (DS), defined as the average number of carboxymethyl groups introduced per AGU. Its theoretical maximum is 3, whereas commercial grades typically fall in the range of approximately 0.4–1.3 [3,10], while the EU specification for the food additive E 466 admits DS values of 0.2–1.5 [8]. DS governs water solubility, pH sensitivity, chemical reactivity, thermal stability, rheology and biodegradability, and it is therefore the primary target of any process-optimisation study [11,12]. Together with molecular weight—which principally controls solution viscosity—and with the distribution of the substituents along and among the chains, DS defines the application profile of a given grade [13,14]. Because DS and molecular weight are, to a first approximation, independent variables, the two can be tuned separately: DS by the etherification stoichiometry and reaction conditions, and molecular weight by the choice of parent cellulose and the extent of chain degradation during processing. This decoupling is central to the present study, which characterises both descriptors across a large sample of waste feedstocks.

Industrially, CMC is manufactured by the alkali-catalysed Williamson etherification of cellulose with monochloroacetic acid (MCA) or its sodium salt (MCA-Na). The reaction proceeds in two stages. In the first, “alkalisation” or “mercerisation”, cellulose is swollen and activated with aqueous sodium hydroxide to form reactive alkali cellulose (Cell–O^−^Na^+^); this step disrupts the crystalline hydrogen-bond network and improves reagent accessibility. In the second, etherification, the alkali cellulose reacts with the chloroacetate to yield the carboxymethyl ether, with sodium chloride and—via a competing hydrolysis of MCA—sodium glycolate as the principal by-products [15,16]. The reaction is almost always carried out heterogeneously in an alcohol–water slurry (isopropanol or ethanol), which moderates the exotherm, limits the hydrolytic side reaction and improves the uniformity of substitution relative to purely aqueous media [15,16]. Reaction temperature, alkali-to-cellulose and MCA-to-cellulose molar ratios, solvent composition and water content, and the sequencing of reagent addition are the levers most frequently reported to control DS, purity and molecular weight [16,17].

Interest in alternative, low-cost cellulose feedstocks for CMC production has grown rapidly, driven both by the price of purified cotton linters and bleached wood pulp and by the imperative to valorise waste streams [17,18]. CMC has been synthesised successfully from cotton gin waste, with a reported DS of 0.87 obtained at an optimal 20% NaOH concentration [17]; from office and disposable-paper waste, reaching DS values of 1.07 and 1.21, respectively [18,19]; and from a range of agro-industrial residues including sugar-beet pulp, rice straw, corn husks, banana pseudostem, durian rind and sugarcane bagasse [12,20,21]. Bacterial cellulose has also been exploited as a highly pure, tailorable substrate [20]. Most relevant to the present work, several groups have targeted textile wastes specifically: Rahaman et al. produced technical, semi-purified and purified grades from waste textile cotton with DS of 0.56, 0.79 and 1.00 and purities of 62, 79 and 99%, respectively [10]; Islam and Mondal obtained DS from 0.90 to 2.84 through one- to seven-step carboxymethylation of textile rags [22]; and Bidgoli et al. converted cotton and viscose waste textiles into cross-linked superabsorbents with DS of 0.50–0.86 and water-binding capacities exceeding 500 g g^−1^ [23]; more recently, post-consumer denim was carboxymethylated in situ after high-energy milling, which lowered the crystallinity index of the recycled cellulose from 75.7 to 66.1% and made it a more reactive substrate [24]. These studies show that waste-derived cellulose can be carboxymethylated to products comparable with those obtained from conventional feedstocks, although usually from a single, pre-cleaned waste stream.

The environmental and economic motivation for this route is strong. Global fibre production reached roughly 124 million tonnes in 2023 and is projected to approach 145–160 million tonnes by 2030 [25,26,27]. Cotton alone accounts for a large share of the natural-fibre fraction, and its cellulosic content makes end-of-life cotton garments an attractive secondary raw material. Yet the textile sector remains strikingly linear: of the roughly 92 million tonnes of textile waste generated annually, only about 15% is collected for reuse or recycling and merely around 1% is recycled fibre-to-fibre, while more than 70% is landfilled or incinerated [27,28,29]. Textile manufacture and disposal rank among the leading contributors to water pollution, greenhouse-gas emissions and microplastic release, and the average garment is now used for some 40% less time before disposal than two decades ago [26,29]. Converting the cellulosic fraction of discarded garments into a high-value chemical such as CMC exemplifies the material-recovery strategy at the heart of the circular economy, keeping carbon in productive use, deferring final disposal and substituting virgin feedstock [30,31,32]. The regulatory context is also changing rapidly: separate collection of textiles has been mandatory in all EU Member States since 1 January 2025, and Directive (EU) 2025/1892 introduces harmonised extended producer responsibility schemes that oblige producers to finance the collection, sorting and recycling of used textiles [33]. These measures will substantially increase the volume of collected post-consumer textiles, a large share of which is unsuitable for reuse or fibre-to-fibre recycling and therefore requires complementary, chemically based recovery routes [27,29].

At the same time, the demand landscape for CMC is broadening beyond its traditional markets. Water-soluble, fluorine-free CMC binders are increasingly adopted in lithium-ion battery electrodes and separators, where they replace poly(vinylidene fluoride) dissolved in N-methyl-2-pyrrolidone (PVDF/NMP) and thereby remove a hazardous solvent from cell manufacture while improving aqueous processability and adhesion [34,35,36]. CMC is particularly effective in silicon- and graphite-based anodes, where its rigid, carboxylate-rich backbone buffers the large volume changes of the active material and stabilises the solid-electrolyte interphase [34,35].

Despite these advances, the published literature is dominated by studies of a single, carefully purified waste stream processed under narrowly optimised conditions. A practical recycling operation, by contrast, must cope with heterogeneous, coloured, blended and contaminated feedstocks that arrive as reference fabrics, pre-consumer clippings and post-consumer garments, and it must do so with a single, robust process. Product quality across such feedstock heterogeneity and the effect of synthesis route have not been assessed systematically across a large, deliberately heterogeneous panel. The present work addresses this gap directly. We carried out fifty-five carboxymethylation experiments on cotton, viscose, flax, jute and blended textile wastes drawn from three provenance classes, using five synthesis variants, and evaluated the products for DS, viscosity, purity, pH and moisture. The data were analysed statistically by one-way analysis of variance (ANOVA), Tukey honestly significant difference (HSD) post hoc testing and correlation/regression analysis to quantify associations between waste provenance, process route and CMC quality. The overarching objective was to determine how a heterogeneous textile-waste panel responds to a common carboxymethylation platform and to assess its capacity to produce technical-grade CMC with tunable viscosity. The overall experimental workflow and reaction scheme are shown in Figure 1.

## 2. Materials and Methods

The raw materials, reagents, apparatus and analytical procedures used in this study are described below. All syntheses were performed at laboratory scale in a solvent slurry; feedstocks were pre-shredded to defined particle sizes prior to reaction.

### 2.1. Feedstocks

Cellulose-bearing textile wastes were assigned to three provenance classes: reference fabrics (symbol S)—commercial woven fabrics and raw bast fibres used as controls; pre-consumer waste (symbol K)—sewing-room clippings and offcuts; and post-consumer waste (symbol P)—used garments and household textiles. The panel was designed to reflect the input actually received by a textile-recycling operation rather than an idealised single stream: cotton is the dominant cellulosic fibre in both pre- and post-consumer waste, viscose is the most important man-made cellulosic fibre, flax and jute represent bast fibres that still contain non-cellulosic components (hemicellulose, lignin, pectins), and cotton–elastane, cotton–flax and viscose–cotton blends represent the multi-component garments that are the most difficult to recycle fibre-to-fibre [29,37]. Pre-consumer clippings (single composition, no use phase) and post-consumer garments (dyes, finishes, detergent residues, mechanical wear) were included specifically to test whether the use phase affects carboxymethylation and product purity, and the microcrystalline cellulose served as a contaminant-free internal reference. Raw flax (100%) and raw jute (100%) were supplied by VIVE Innovation Sp. z o.o. (Kielce, Poland), one of the largest textile-recycling companies in Central Europe. Cotton fabrics (100% cotton, 125–150 g·m^−2^, various colours) were obtained from Fashion Colours (Wola Rębkowska, Poland); cotton, cotton–elastane, cotton–flax and viscose–cotton blends were obtained from PPHU TEONA (Krotoszyn, Poland). Pre-consumer clippings originated from sewing plants in Radom, Poland, and were milled industrially. Post-consumer items (dresses, trousers, baby bodysuits, T-shirts, pillowcases, bed sheets, flannel shirts) of 100% cotton, cotton–elastane, cotton–flax and 100% viscose were supplied by VIVE Innovation. A microcrystalline reference cellulose (Arbocel B400, J. Rettenmaier & Söhne GmbH + Co KG, Rosenberg, Germany) was included as an internal control. The compositions of the feedstocks are summarised in Table 1. The fibre compositions of the commercial fabrics and post-consumer items are those declared by the suppliers or printed on the garment care labels before milling.

### 2.2. Reagents

The following reagents were used: monochloroacetic acid (MCA, 80% HP and 100% technical flakes; PCC MCAA Sp. z o.o., Brzeg Dolny, Poland); sodium monochloroacetate (MCA-Na, for synthesis; Sigma-Aldrich, Inc., St. Louis, MO, USA); propan-2-ol (isopropanol, analytical grade; STANLAB Sp. z o.o., Lublin, Poland); ethanol (96% analytical grade, STANLAB Sp. z o.o., Lublin, Poland; and 96% denatured, Linegal Chemicals Sp. z o.o., Blizne Łaszczyńskiego, Poland); sodium hydroxide (1 mol·L^−1^ standardised, AlfaChem Sp. z o.o., Poznań, Poland; 50% analytical grade, Chempur, Piekary Śląskie, Poland; microgranules, POCH S.A., Gliwice, Poland); hydrochloric acid (35–38% analytical grade, Chempur, Piekary Śląskie, Poland; 1 mol·L^−1^ standardised, AlfaChem Sp. z o.o., Poznań, Poland); dichloroacetic acid (DCA, 98.0%, Merck KGaA, Darmstadt, Germany); hydrogen peroxide (30%, POCH S.A., Gliwice, Poland); nitric acid (65%), sulfuric acid (95%) and methanol (99.8%). Solvent mixtures were prepared with deionised water.

### 2.3. Apparatus

Textile wastes were size-reduced in two stages. Laboratory-scale material was pre-cut to 5–10 mm squares and milled in an IKA MF 10 B cutting mill (IKA-Werke GmbH & Co. KG, Staufen, Germany; 4500–5500 min^−1^) or a Testchem 2S/El 90 L2 knife mill (Testchem Sp. z o.o., Radlin, Poland; 2800 min^−1^). Pilot-scale shredding was performed by industrial pulverisers from Pallmann Maschinenfabrik (Zweibrücken, Germany), Herbold Meckesheim GmbH (Meckesheim, Germany) and Hosokawa Alpine AG (Augsburg, Germany), producing final sieve fractions of 0.5, 1.0 or 2.0 mm. Syntheses were carried out in a 500 mL three-neck round-bottom flask fitted with a reflux condenser, mechanical stirrer, thermometer, dropping funnel and water bath. Portions of the crude products were purified by Soxhlet extraction and used for the preparation of the viscosity solutions (Section 2.5). Viscosity was measured with Brookfield DV-II+ (Brookfield Engineering Laboratories, Inc., Middleboro, MA, USA) and Anton Paar ViscoQC 300 R rotational viscometer (Anton Paar GmbH, Graz, Austria) and a Höppler BH2 falling-ball viscometer; moisture was determined with a Radwag MAC 210 moisture analyser (RADWAG Wagi Elektroniczne, Radom, Poland) (infrared, 120 °C); masses were recorded on Radwag analytical balances (RADWAG Wagi Elektroniczne, Radom, Poland).

### 2.4. Synthesis Routes

Five synthesis routes were evaluated, all sharing the same alkalisation–etherification sequence but differing in reagent-addition strategy or additives. Method I (baseline, single-step): isopropanol and water were charged to the flask, followed by the shredded feedstock; after 10 min of stirring, aqueous NaOH (50%) was added dropwise and the slurry was alkalised at room temperature (20 °C) for 30 min. MCA solution was then added dropwise and the flask was transferred to a 60 °C water bath; aliquots were withdrawn at 45 and 90 min, and the reaction was terminated 120 min after acid addition. Each aliquot was neutralised with 1 N HCl to the phenolphthalein end-point and used only for pH and viscosity measurements; DS was determined exclusively on the final (120 min) product. Method II (stepwise reagents): NaOH was added in two portions 30 min apart before etherification, to promote more uniform alkali-cellulose formation. Method III (bleaching/degrading): 30% H_2_O_2_ was introduced immediately after adding NaOH to bleach coloured feedstocks and partially degrade the cellulose chain, lowering molecular weight. Method IV (cross-linking): dichloroacetic acid (DCA, 0.35 mL) was co-dosed with MCA as a bifunctional cross-linker. Method V (sodium-salt etherification): solid MCA-Na was added in small portions instead of MCA solution. Reactions were run at feedstock: NaOH:MCA molar ratios (referred to the glycosidic fragment) of typically 1:2.30:1.15, with variants at 1:1.50:0.75 and 1:3.00:1.50, and with a doubled NaOH charge in a few runs (Section 3.2); solvent volume and alkalisation/etherification times were varied as specified in Table S1.

All syntheses were carried out on 12.0 g of air-dry shredded feedstock. The reagent charges were calculated on this air-dry mass taken as anhydroglucose units (12.0 g/162.14 g·mol^−1^ = 0.074 mol AGU), i.e., without correction for moisture (typically 6–8% in air-dry textiles) or for non-cellulosic components, so that the actual molar ratios per AGU were somewhat higher than the nominal values. The feedstock was dispersed in 231.25 mL of isopropanol and 18.75 mL of water (isopropanol:water 92.5:7.5, *v*/*v*; liquor ratio ≈ 21 mL·g^−1^); in some runs the solvent volume was increased to 323.75 mL of isopropanol and 26.25 mL of water at the same isopropanol:water ratio (≈29 mL·g^−1^), as specified for each run in Table S1. At the baseline ratio of 1:2.30:1.15 the nominal charge corresponds to 6.81 g of NaOH (13.6 g of the 50% solution) and 8.04 g of MCA (100% basis; 10.1 g of the 80% grade) or, in Method V, 9.91 g of MCA-Na. Including the water introduced with the 50% NaOH solution and with the 80% MCA grade, the liquid phase contained about 12–13% (*w*/*w*) of water at the start of etherification. At the mean DS of 0.48, the theoretical mass of Na-CMC is 14.8 g per batch (repeat-unit molar mass 200.5 g·mol^−1^), and at the mean purity of the reference products (75.9%), the corresponding dry crude product amounts to ≈19.6 g. This quantity is sufficient for yield determination and for the complete analytical programme performed on each run: DS determination (≈4 g of crude product for acid conversion), purity (3 g), preparation of the 2% solution for viscosity and pH (≈4.8 g of polymer for 240 g of solution) and moisture (1–2 g), leaving a retained sample for repeat analyses. The aliquots withdrawn at 45 and 90 min (≈10 mL of slurry each) were used only for in-process monitoring of pH and viscosity and were excluded from the yield calculation.

### 2.5. Characterisation

The degree of substitution was determined by the acid-wash/back-titration method (ASTM D1439, Test Method A [38]). The dried crude product was converted to the free-acid (H-CMC) form, a known mass was suspended and treated with a measured excess of standardised NaOH, and the unreacted alkali was back-titrated with standardised HCl. DS was calculated from the consumed alkali according to the standard relationship DS = (0.162·A)/(1 − 0.058·A), where A is the milliequivalents of carboxymethyl groups per gram of sample. Active-substance content (purity, %) was determined gravimetrically as the fraction of true CMC in the crude product after removal of sodium chloride and sodium glycolate by-products. The 2% (*w*/*w*) apparent viscosity was measured at 20 °C; residual moisture was measured by infrared drying; and pH was measured with the 2% aqueous solution.

Acidification and washing were performed as follows. Approximately 4 g of the crude product was slurried in 75 mL of 95% ethanol under mechanical stirring, 5 mL of concentrated nitric acid was added, and stirring was continued for 1–2 min; the slurry was then boiled for 5 min and stirred without heating for a further 10–15 min to convert Na-CMC quantitatively into the water-insoluble free-acid form (H-CMC). The supernatant was decanted through a fritted-glass funnel; the precipitate was transferred with 50–100 mL of 95% ethanol and washed with 80% (*v*/*v*) ethanol pre-heated to 60 °C until the washings were free of nitrate, as verified by the absence of a blue colour on a spot test with diphenylamine reagent (0.5 g of diphenylamine in 120 mL of H_2_SO_4_, 9 + 2); two to three washings were normally required. This washing sequence removes the excess acid together with sodium chloride and sodium glycolate. The H-CMC was finally rinsed with a small volume of anhydrous methanol, freed from alcohol, dried at 105 °C for 3 h and cooled in a desiccator. A 1.0–1.5 g portion of the dry H-CMC was dispersed in 100 mL of deionised water, 25.00 mL of 0.4 mol·L^−1^ NaOH was added, and the mixture was boiled for 15–30 min to reconvert the acid groups into the sodium salt; the excess alkali was titrated while hot with 0.4 mol·L^−1^ HCl to the phenolphthalein end-point. Both titrants were prepared by dilution of the standardised 1 mol·L^−1^ solutions (Section 2.2). A (meq·g^−1^) was calculated as A = (V_NaOH·c_NaOH − V_HCl·c_HCl)/m_H-CMC. DS was generally determined by a single titration; a duplicate determination was made when the first result appeared inconsistent, and the mean of the two values was then reported. Test Method A is specified for crude CMC with DS up to about 0.85, which covers all products of this study, and its repeatability is ±0.04 DS units at the 95% confidence level [38]. Because the method titrates polymer-bound carboxyl groups, the calculated DS reflects the total carboxyl functionality retained on the polymer after each synthesis route. Sodium chloride, sodium glycolate and unreacted chloroacetic acid are removed by washing the water-insoluble H-CMC with 80% ethanol before titration; the titratable acidity measured in this procedure therefore originates from polymer-bound carboxyl groups and, together with the water solubility of the products, provides chemical evidence of carboxymethylation.

Purity (active-substance content). Throughout this work, “crude product” denotes the neutralised and dried reaction product, which contains Na-CMC together with the sodium chloride and sodium glycolate by-products and any non-cellulosic matter carried over from the feedstock. The purity values in Table 2 and Table 3 and in Figure 2 and Figure 3 always refer to this crude product and are expressed on a dry basis; the Soxhlet-extracted material mentioned in Section 2.3 was used only for the preparation of the viscosity solutions and not for the reported purity values. Purity was determined gravimetrically according to ASTM D1439 (purity of crude sodium carboxymethylcellulose) [38]. A 3.0 ± 0.1 g portion of the crude product, weighed to ±1 mg, was stirred in a covered 400 mL beaker with 150 mL of 80% (*v*/*v*) ethanol (specific gravity 0.857 ± 0.001) at 60–65 °C for 10 min in a thermostatted water bath; the undissolved polymer was allowed to settle, and the hot supernatant containing the dissolved salts was decanted through a tared, medium-porosity fritted-glass crucible. The extraction was repeated with a second 150 mL portion of hot 80% ethanol, after which the residue was transferred quantitatively to the crucible with further hot 80% ethanol (≈250 mL in total), washed with 50 mL of 95% ethanol at room temperature and dried at 105 ± 1 °C to constant mass (change ≤ 3 mg per 1 h drying period). The moisture content of the crude product (C, %) was determined on a separate portion, and purity was calculated as S = 10,000·A/[B·(100 − C)], where A is the mass of the dried residue (g) and B the mass of the specimen (g). Purity was generally determined once per product; a second determination was made when the first result appeared doubtful, and the mean of the two values was then reported. ASTM D1439 reports interlaboratory reproducibility of ±0.6 percentage points at the 95% confidence level [38]. Because the ethanol-insoluble residue also retains insoluble non-cellulosic matter, the reported value for blended feedstocks represents the total ethanol-insoluble fraction remaining after extraction.

Viscosity. The 2% (*w*/*w*) solutions were prepared on a dry-polymer basis calculated from the moisture content by adding the Soxhlet-extracted product slowly to deionised water under mechanical stirring (≈500 min^−1^ for 2 h) to avoid agglomeration, followed by equilibration in a thermostatted bath at 20.0 ± 0.2 °C for 1 h; apart from the temperature, the procedure follows ASTM D1439 [38]. Apparent viscosity was measured with rotational viscometers at 20, 30 and 50 min^−1^: solutions of low viscosity with the Brookfield DV-II+ fitted with the LV-1 spindle and solutions of higher viscosity with the Anton Paar ViscoQC 300 R; the spindle was selected so that the torque reading was as close as possible to 50% and, whenever possible, within 10–90% of the full-scale range. For the lowest-viscosity products (below about 12 mPa·s, corresponding to Brookfield torque below 10% of full scale at 50 min^−1^), the dynamic viscosity was additionally measured with the Höppler BH2 falling-ball viscometer as a supplementary check; these values were not used in the statistical analysis. The apparent viscosities reported in Table 2 and Table 3 and used in the statistical analysis were obtained at 50 min^−1^. Supplementary Table S1 identifies the final-product viscosity used for each run; the 20 and 30 min^−1^ readings were treated as auxiliary rheological checks. Because the solutions are shear-thinning, apparent viscosity depends on measurement geometry and shear rate [38]; standardising the reported dataset to 50 min^−1^ provides a consistent basis for comparison and grade classification. All statistical analyses were performed on log_10_(η). The ±10–20% inter-instrument range used for method-level comparison corresponds to about ±0.04–0.08 log units, substantially smaller than the within-class standard deviations of 0.79–1.20 log units (Table 3).

### 2.6. Statistical Analysis

Each synthesis was treated as an independent observation (*N* = 55). Each run was a separately weighed, alkalised and etherified batch representing a distinct combination of feedstock, synthesis route and reagent ratio. ASTM D1439 repeatability values (±0.04 DS units for Test Method A and ±0.6 percentage points for purity, both at the 95% confidence level) were used as method-level precision references [38]. Descriptive statistics (mean ± standard deviation) were computed per feedstock class. Differences among the three provenance classes (S, K, P) in DS, purity and log_10_-transformed viscosity were tested by one-way analysis of variance (ANOVA); viscosity was log-transformed because it spanned four orders of magnitude and was strongly right-skewed. Where ANOVA indicated significance (*p* < 0.05), Tukey’s honestly significant difference (HSD) test was used for pairwise comparisons. The effect of synthesis route on DS was tested by ANOVA restricted to methods with *n* ≥ 4 (I, III, V). Pairwise linear relationships among DS, purity and log-viscosity were quantified by Pearson correlation coefficients and ordinary least-squares regression. Analyses were performed in Python 3.12 (Python Software Foundation, Wilmington, DE, USA) using the SciPy and statsmodels libraries.

The 55-run comparative dataset was selected from a laboratory process-development programme comprising 111 syntheses (Method I, 72 runs; Method II, 1; Method III, 6; Method IV, 1; Method V, 7; and 24 runs of a further variant not considered in this study). The programme intentionally covered variation in feedstock provenance, fibre composition, particle size, synthesis route and reagent ratio. The analysed dataset comprised 41 runs in Method I, 6 in Method III, 6 in Method V and one each in Methods II and IV; all pre-consumer feedstocks listed in Table 1 were 100% cotton. Provenance and route ANOVAs were used to compare the observed groups, with the detailed run structure reported to support interpretation of the group-level results. The comparative dataset comprised the predefined cross-provenance series I.1–I.21 plus I.25, I.27 and I.30–I.32; II.2–II.4 and II.6–II.10; and III.1–III.21. These 55 runs constituted the planned comparative series and contained the complete response set required for the present analysis (DS, final 2% viscosity, purity, pH and moisture); the Method VII time-optimisation series and other process-development runs were outside this comparison.

The assumptions of the parametric analyses were examined. Normality of the ANOVA residuals was assessed with the Shapiro–Wilk test and normal Q–Q plots, homogeneity of variances with Levene’s test, and influential observations in the regressions with Cook’s distance (threshold 4/*N*). Shapiro–Wilk tests of the class-model residuals gave W = 0.863, *p* < 0.001 for DS; W = 0.840, *p* < 0.001 for purity; and W = 0.940, *p* = 0.009 for log_10_(η). Levene’s tests gave F(2,52) = 0.533, *p* = 0.590 for DS; F(2,52) = 1.615, *p* = 0.209 for purity; and F(2,52) = 1.976, *p* = 0.149 for log_10_(η). With the Cook threshold 4/*N* = 0.0727, 5, 4 and 2 observations exceeded the threshold in the DS–purity, DS–log_10_(η) and purity–log_10_(η) regressions, respectively. Because the groups were unbalanced and the post-consumer purity distribution was visibly broader, Welch’s heteroscedasticity-robust ANOVA with Games–Howell post hoc comparisons was applied as a conservative robustness analysis in addition to the classical ANOVA and Tukey HSD test. Kruskal–Wallis tests were also used as a distribution-free check and were non-significant for provenance effects on DS (H = 1.82, *p* = 0.402), purity (H = 4.81, *p* = 0.090) and log_10_(η) (H = 0.42, *p* = 0.812). Spearman rank correlation coefficients were computed alongside the Pearson coefficients to verify that the correlations were not driven by a few extreme observations (DS–purity, ρ = 0.538, *p* < 0.001; DS–log_10_(η), ρ = 0.432, *p* = 0.001; purity–log_10_(η), ρ = 0.672, *p* < 0.001). To examine whether the absence of a significant provenance effect on DS is compatible with practical equivalence, two one-sided tests (TOST, Welch-corrected) were performed for each pair of classes with pre-specified equivalence margins of ±0.05 and ±0.10 DS units; the narrower margin is close to the repeatability of the titrimetric DS determination (±0.04 at the 95% confidence level [38]), and the wider one corresponds to about half the width of the DS window typically quoted in commercial CMC specifications. Statistical power was expressed as the minimum difference detectable with 80% power at α = 0.05. Welch’s ANOVA, Games–Howell comparisons, TOST and power were calculated from the class means, standard deviations and sample sizes in Table 3.

### 2.7. Scanning Electron Microscopy and EDS Microanalysis

The morphology and surface elemental composition of selected shredded cotton feedstocks were examined by scanning electron microscopy (SEM) with energy-dispersive X-ray spectroscopy (EDS). Imaging was performed on an Apreo 2S LoVac field-emission microscope (Thermo Fisher Scientific, Waltham, MA, USA) equipped with UltraDry (Thermo Fisher Scientific, Waltham, MA, USA) and Octane Elect (EDAX, Inc., Mahwah, NJ, USA) EDS detectors. All micrographs and spectra were acquired at an accelerating voltage of 2 kV using the T1 in-lens detector (A+B mode). Prior to analysis, the samples were conditioned to constant mass at 23 ± 2 °C and a relative humidity not exceeding 50 ± 5%, in accordance with ISO 291 [39]. Representative pre-consumer (K1) and post-consumer (P2, P18) cotton feedstocks were imaged over the 500–20,000× magnification range; per-sample EDS returned atomic and mass fractions together with net X-ray counts. The measurements provide controlled near-surface characterisation of the selected feedstocks.

## 3. Results

A representative subset of the process conditions and product characteristics is given in Table 2, and the complete dataset for all fifty-five syntheses—feedstock, composition, synthesis route, reagent ratios, process conditions and all measured responses—is provided in Supplementary Table S1. Products covered a wide property space: DS from 0.17 to 0.60, 2% apparent viscosity from 2.5 to 23,100 mPa·s, purity from 52 to 81%, pH from 5.7 to 11.2 and residual moisture from 1.4 to 12.8%. The overwhelming majority of products dissolved to clear or lightly coloured solutions, consistent with successful carboxymethylation (see also the acid-wash titration evidence, Section 2.5); only a few blended or heavily contaminated feedstocks (e.g., flannel P12c, some cotton–elastane items) gave turbid, sedimenting dispersions and correspondingly low DS. For these products, the viscosity and pH values describe the partly dispersed state of the material. Qualitatively, the residual dye of coloured garments carried through to the CMC solution, giving tinted but otherwise transparent products, whereas bleaching with H_2_O_2_ (Method III) yielded pale, low-viscosity products. Colour and non-cellulosic contaminants therefore affected appearance and purity more strongly than the measured substitution level.

### 3.1. Degree of Substitution Across Feedstock Classes

The mean DS across all products was 0.48 ± 0.07. Class means were similar: 0.483 ± 0.082 for reference fabrics (S, *n* = 26), 0.469 ± 0.038 for pre-consumer waste (K, *n* = 8) and 0.468 ± 0.064 for post-consumer waste (P, *n* = 21) (Table 3). One-way ANOVA found no significant effect of feedstock class on DS (F(2,52) = 0.33, *p* = 0.72), and all Tukey HSD pairwise comparisons were non-significant (*p* > 0.72). Thus, no statistically significant difference in DS between the provenance classes was detected under the conditions investigated (Figure 2a). It is worth emphasising that the pre-consumer class showed not only a comparable mean but also the smallest scatter (SD 0.038), reflecting the relative cleanliness and homogeneity of sewing-room clippings, which are single-composition offcuts free of dyes and finishing agents. This observation agrees with recent studies identifying the purity and accessibility of the parent cellulose as primary determinants of carboxymethylation: appropriate hydrogen-peroxide delignification increased the DS of CMC prepared from palm bunch and bagasse [12], cellulose isolated from matchstick waste by an ionosolv pretreatment gave CMC with a DS of 2.3 and a purity of 98.5%, clearly superior to the product from conventionally extracted cellulose [40], and refining or enzymatic treatment of bleached pulp raised the DS from 0.5 to 2.4 by improving the accessibility of the hydroxyl groups [41]. The post-consumer class, while similar in mean DS, exhibited the widest range (0.31–0.57), driven by a small number of heavily blended items; excluding the two contaminated flannel/blend outliers, the post-consumer mean rises marginally and its variance contracts, consistent with similar reactivity of clean cotton garments and reference fabric.

The absence of a detectable DS effect was robust to unequal class variances (Welch’s ANOVA, F(2,27.4) = 0.29, *p* = 0.75; all Games–Howell comparisons *p* > 0.7). The equivalence analysis placed all three pairwise class differences within a margin of ±0.10 DS units (TOST, *p* ≤ 0.0002). At the stricter ±0.05 margin, equivalence was supported for the pre- versus post-consumer comparison (*p* = 0.010), while the reference versus pre-consumer and reference versus post-consumer comparisons gave *p* = 0.049 and *p* = 0.054, respectively. With the present group sizes, the minimum detectable difference between the reference and post-consumer means at 80% power was approximately 0.06 DS units.

### 3.2. Product Purity

Purity behaved differently from DS. Class means were 75.9 ± 3.9% (S), 76.1 ± 3.8% (K) and 71.9 ± 7.3% (P), and ANOVA indicated a significant effect of feedstock class (F(2,52) = 3.59, *p* = 0.035). The Tukey HSD test localised the difference to the post-consumer versus reference-fabric contrast (mean difference −4.0 percentage points, *p* = 0.039), whereas pre-consumer waste did not differ significantly from reference fabrics (*p* = 0.998) (Figure 2b). The post-consumer class was also more dispersed (SD 7.3% versus 3.9% for S and 3.8% for K). Levene’s test did not reject homogeneity of variances (F(2,52) = 1.62, *p* = 0.209); Welch’s ANOVA gave F(2,20.4) = 2.67, *p* = 0.093, and the Games–Howell post-consumer versus reference comparison gave *p* = 0.077. Accordingly, the post-consumer group combined a lower mean with a broader distribution. The two lowest-purity products (53–52%, items P3a in Table 2 and P4c in Table S1) were obtained under a doubled-NaOH protocol, which increased the sodium chloride and sodium glycolate by-product load and depressed the measured active-substance fraction; under the baseline reagent ratio, the same feedstocks reached 76–78% (Table S1). Several post-consumer cotton items also reached 77–78% purity, on par with reference fabric, indicating that the lowest values were concentrated in blended and over-alkalised samples.

Purity is a primary specification parameter of CMC rather than a secondary descriptor, and it deserves particular attention when textile waste is the feedstock. First, purity defines the commercial grade: technical (crude) CMC retains the sodium chloride and sodium glycolate formed during the synthesis—Rahaman et al. reported about 62% active substance for technical-grade CMC from waste textile cotton [10]—whereas CMC used as the food additive E 466 must contain at least 99.5% Na-CMC on an anhydrous basis [8,9]. Second, the by-products are not inert: sodium chloride screens the carboxylate charges and lowers the viscosity of Na-CMC solutions, particularly at low polymer concentrations [13,42], and for this reason the viscosity solutions in this study were prepared from Soxhlet-extracted product (Section 2.5). Third, for post-consumer textiles, purity integrates contributions that are absent from virgin pulp—dyes, finishing resins, detergent and softener residues, mineral particles and synthetic fibres—and therefore serves as a sensitive indicator of feedstock cleanliness and of the effectiveness of pre-sorting. Finally, the gravimetric method retains insoluble non-cellulosic matter, including synthetic fibres, in the residue, so the purity of products from blended feedstocks represents the combined ethanol-insoluble fraction (Section 2.5 and Section 4). It was precisely to quantify these effects that wastes of different provenance and composition were selected (Section 2.1): purity, rather than DS alone, decides for which grade and application a textile-waste-derived CMC can be qualified.

### 3.3. Solution Viscosity

Apparent viscosity of the 2% solutions varied over four orders of magnitude. At this concentration, apparent viscosity reflects the combined effects of molecular weight, DS, substituent distribution, residual salt content and chain association [13,14,42], and it is therefore treated here as the directly measured rheological descriptor. Class means of the raw viscosity were dominated by a few very high-viscosity samples (e.g., flax S14c, 23,100 mPa·s; cotton garment P11a, 14,300 mPa·s), and ANOVA on log-transformed viscosity showed no significant class effect (F(2,52) = 0.22, *p* = 0.81) (Figure 4). This wide, feedstock-independent viscosity range is practically useful: by selecting the feedstock and synthesis route, the same process delivered both low-viscosity grades (<50 mPa·s, suitable as dispersants or flow modifiers) and high-viscosity grades (>5000 mPa·s, suitable as thickeners or binders). Such a wide range is characteristic of CMC in general: commercial low-, medium- and high-viscosity grades differ in consistency by more than two orders of magnitude at the same concentration, mainly as a consequence of molecular weight [43], and in industrial practice the viscosity grade is set primarily by the degree of polymerisation of the starting cellulose—cotton linters for high and wood pulp for medium molar masses—while low-viscosity grades are obtained by deliberate depolymerisation [42]. For waste-derived CMC, Rahaman et al. reported 501–4160 mPa·s for technical to purified grades obtained from waste textile cotton [10], and Suriyatem et al. showed that the extent of H_2_O_2_ bleaching of the parent cellulose influences the viscosity of the resulting CMC [12]. The span recorded here reflects the rheological diversity of a single textile-waste panel and demonstrates that one process can cover a broad viscosity window. Method III (H_2_O_2_) consistently produced low-viscosity products (2.5–30 mPa·s) while leaving DS essentially unchanged. The bast fibres (flax, jute) and the least-degraded cotton garments delivered the highest viscosities, matching the high-viscosity end of the measured range. For a given feedstock, the 120 min viscosity often fell below the 90 min value (Table S1), demonstrating a time-dependent decrease in apparent viscosity and providing an additional process handle on the final grade.

### 3.4. Effect of Synthesis Route

Restricting the analysis to routes with *n* ≥ 4, the mean DS was 0.48 for Method I, 0.42 for Method III and 0.48 for Method V. ANOVA found no statistically significant effect of synthesis route on DS (F(2,50) = 2.12, *p* = 0.13), and Tukey HSD confirmed that no pair differed significantly (Figure 5). Method III (H_2_O_2_) combined the lowest mean DS with consistently low viscosity, whereas Method V matched the baseline DS and generally produced higher viscosities, a useful combination for thickener grades; Method V also avoids handling liquid MCA. The stepwise-reagent (Method II) and cross-linking (Method IV) variants, each represented by a single run and excluded from the ANOVA, gave DS values of 0.45 and 0.57, respectively. Across all five variants, the observed DS values remained within a relatively narrow band, while route selection had a larger practical impact on colour, viscosity and network structure than on mean DS.

The moderate DS obtained across the routes is consistent with the reaction stoichiometry and the known competition between etherification and chloroacetate hydrolysis. At the baseline AGU:NaOH:MCA ratio of 1:2.30:1.15, the theoretical DS ceiling is 1.15, and the mean DS of 0.48 corresponds to an apparent reagent-incorporation efficiency of about 42%. A NaOH:MCA ratio near 2.0 provides little alkali reserve after neutralisation and etherification, while the water introduced with the 50% NaOH and aqueous MCA solutions (about 12–13% *w*/*w* of the liquid phase, Section 2.4) promotes the competing hydrolysis of MCA [16,41]. Method V provides a larger nominal alkali reserve because MCA-Na requires no neutralisation, yet its mean DS remained 0.48, showing that alkali availability, chloroacetate hydrolysis and cellulose accessibility act together in determining substitution. The literature data show that multi-step carboxymethylation and optimisation of alkali concentration, etherifying-agent charge and reaction time increase DS in waste-derived celluloses [17,18,22]. These relationships identify direct process levers for moving DS toward the 0.7–0.9 range used in many CMC applications [3]: a higher MCA:AGU ratio, increased NaOH:MCA ratio, lower slurry water content and/or a second etherification stage.

### 3.5. Structure–Property Correlations

Pairwise correlation analysis (Figure 6) revealed statistically significant relationships. DS correlated positively with purity (r = 0.38, *p* = 0.004) and with log-viscosity (r = 0.42, *p* = 0.002), and purity correlated with log-viscosity (r = 0.57, *p* < 0.001). The DS–purity regression, DS = 0.0047·(purity) + 0.13 (R^2^ = 0.15, *p* = 0.004), quantifies the positive association between these descriptors (Figure 3). Purity accounted for about 15% of the variance in DS, while the stronger purity–log-viscosity correlation identified viscosity as the response most closely associated with active-substance content in this dataset. pH and moisture were weakly and non-significantly related to the primary descriptors, consistent with their control by final neutralisation and drying rather than by substitution chemistry.

### 3.6. Fibre Morphology and Surface Composition (SEM/EDS)

Representative shredded cotton feedstocks were imaged to relate the statistical purity trend (Section 3.2) to the physical state of the fibres. The pre-consumer sewing-room cotton (K1) presented smooth, ribbon-like, twisted fibres with clean surfaces and largely intact ends, consistent with single-composition offcuts that have not been through a use phase (Figure 7a). The post-consumer cottons (P2, P18) were visibly more degraded: their fibres showed frayed and splayed ends, longitudinal splitting, surface roughening and adhering debris (Figure 7b,c), and at higher magnification discrete bright particulate deposits were resolved on the fibre surfaces (Figure 7d). This morphological contrast—clean pre-consumer versus abraded, debris-bearing post-consumer fibre—matches the direction of the lower and more variable purity measured for post-consumer CMC.

The EDS data in Table 4 characterise the near-surface region of the raw feedstock fibres. At an accelerating voltage of 2 kV, the electron penetration depth in a low-density organic matrix such as cellulose is of the order of 100–150 nm, so the analysis reports the outer fibre layer and adhering particles. At 2 kV, the K shell of sulfur (critical excitation energy 2.47 keV) is not ionised, and the Al K shell (1.56 keV) is excited at a low overvoltage ratio (≈1.3); accordingly, the S and Al values are used as indicators of presence rather than as quantitative concentrations. Aluminium was detected at 0.1–0.2 at.% on the pre-consumer cottons (K1a, K2a), while P18b showed a locally elevated value of 0.6 at.%; sulfur signals were recorded for P2b and P18a,b. The B and N signals were omitted from the interpretation because of peak overlap with the C K peak at these low energies. These surface signatures accompany the more debris-rich morphology observed for the post-consumer fibres.

## 4. Discussion

The DS values obtained here (0.17–0.60, mean 0.48) are consistent with the lower-to-middle range reported for single-step carboxymethylation of waste-derived cellulose and show that a heterogeneous textile-waste panel can be processed to predominantly water-soluble, technical-grade CMC without feedstock pre-selection. Rahaman et al. reported DS of 0.56 for technical-grade CMC from waste textile cotton, rising to 0.79 and 1.00 after semi-purification and purification, respectively [10]; our single-step, unpurified products sit squarely within their technical-grade regime. Similarly, Mondal and co-workers obtained DS of 0.90 only after a fourth carboxymethylation step, with the first step delivering values comparable to ours [22]. Haleem et al. reached DS 0.87 from cotton gin waste but only after scouring, bleaching and optimising the NaOH concentration to 20% [17], and Joshi et al. achieved DS 1.07 from deinked office-paper pulp under carefully controlled conditions [18]. Recent work on textile and other waste celluloses points in the same direction: moderate DS values are typical of single-stage processing of highly ordered cellulose, and substantially higher values have been reached only after treatments that increase accessibility, such as high-energy milling of post-consumer denim [24] or refining and enzymatic treatment of pulp [41]. The single-step, unpurified nature of our process therefore explains the moderate DS (see also Section 3.4) and identifies multi-step operation, higher reagent ratios and dedicated purification as the obvious levers for higher substitution, at the cost of additional reagents and unit operations.

No statistically significant provenance effect on DS was detected (*p* = 0.72), whereas the post-consumer group showed a lower mean purity and a broader purity distribution (classical ANOVA *p* = 0.035; Welch’s ANOVA *p* = 0.093). This decoupling is practically valuable: substitution remained similar across provenance classes, while purity provided the clearer quality discriminator for post-consumer material. The purity of CMC from waste textile cotton can be raised substantially by washing and extraction, as shown by the semi-purified and purified grades of Rahaman et al. [10]. The central role of cellulose purity and accessibility identified in recent studies on lignocellulosic residues [12,40,41] is reflected here mainly in product purity and in the spread of DS rather than in the mean DS, because cotton and viscose textiles are already largely free of lignin and hemicellulose. These observations are in line with the use of textile waste as a secondary cellulose source advocated in circular-economy analyses of the sector [30,31,32]. To our knowledge, this is the first study to compare DS across such a broad, mixed panel of pre- and post-consumer textile wastes.

The SEM/EDS survey of the feedstocks (Section 3.6) complements the purity comparison. The post-consumer fibres showed greater fraying, splitting and adhering debris, together with sulfur signals and locally elevated aluminium on the fibre surface, whereas the pre-consumer cotton was smoother and cleaner. This surface pattern coincides with the lower and broader purity distribution measured for the post-consumer CMC and provides a direct materials-level distinction between the two feedstock classes.

A further practical point concerns solution viscosity. Although the one-way ANOVA on log-viscosity detected no significant provenance effect across the full panel (Section 3.3), the wide feedstock-driven range contains a clear grade-selection pattern. At comparable degree of substitution, CMC obtained from pre-consumer cotton tended to reach higher 2% viscosities than CMC from post-consumer cotton. This measured viscosity separation positions pre-consumer stock toward high-viscosity grades and post-consumer stock toward moderate- and low-viscosity grades.

The four-orders-of-magnitude viscosity range, uncorrelated with provenance, is the second practically significant outcome. Because DS and molecular weight are largely independent quality axes—DS set by etherification stoichiometry and accessibility, molecular weight set by the parent fibre and by degradation—our process can, in principle, place a product anywhere in the (DS, viscosity) plane by combining feedstock choice with route selection. The deliberate use of H_2_O_2_ (Method III) to depress viscosity while preserving DS is a clear example: it mirrors the controlled oxidative depolymerisation used industrially to produce low-viscosity CMC grades, and is consistent with reports that hydrogen peroxide reduces cellulose molecular weight during bleaching [17,23]. Oxidative or acid depolymerisation is the standard industrial route to low-viscosity CMC grades [42], and the present results show that the same lever operates on textile-derived cellulose, where hydrogen peroxide additionally removes residual dye colour. The bast-fibre feedstocks (flax, jute) and least-degraded cotton garments delivered the highest viscosities, consistent with a high degree of polymerisation of the parent fibres. This tunability is commercially meaningful because CMC is sold by viscosity grade at least as much as by DS; a single, feedstock-agnostic process able to span the grade range is therefore potentially attractive for a recycling operation handling variable input.

Our viscometric observations match the polyelectrolyte behaviour of Na-CMC documented in the rheological literature. CMC solutions are strongly shear- and concentration-dependent, exhibit non-Newtonian pseudoplastic flow, and show viscosity governed jointly by DS, molecular weight and ionic environment [13,14,44,45]. The positive DS–viscosity correlation observed here (r = 0.42) aligns with reports that higher charge density promotes chain expansion and higher intrinsic viscosity at comparable molecular weight [44,46]. Benchabane and Bekkour showed that CMC solutions transition from dilute to entangled regimes with rising concentration, each with a characteristic shear-thinning signature [46], and Chalah et al. showed that the consistency of low-, medium- and high-viscosity Na-CMC grades differs by more than two orders of magnitude at the same concentration [43]; these literature ranges bracket the very wide viscosity span recorded here at a fixed 2% concentration. The measured pH range (5.7–11.2) and its dependence on residual alkali also align with the documented pH sensitivity of CMC rheology and gelation [47]. More recent rheological work has shown that lowering pH promotes interchain association and weak-gel formation in CMC solutions, whereas added NaOH suppresses it [42], providing a relevant framework for interpreting the partly alkaline products obtained here.

From an application standpoint, the viscosity and DS ranges obtained here overlap with those of several established CMC grades. Low-viscosity, moderate-DS CMC is used as a dispersant, flow modifier and mineral-processing depressant [7,44]; medium grades serve as food and pharmaceutical thickeners and stabilisers, where DS in the 0.4–0.9 band is standard and approved for several uses [8,9,11] and purified material contains at least 99.5% Na-CMC [8]; high-viscosity grades function as binders and film formers [3,6]. Potential high-value outlets also include aqueous binders for lithium-ion battery electrodes [34,35,36], where CMC is selected according to defined purity, DS and molecular-weight specifications.

The environmental logic of the route deserves emphasis. Because only a small fraction of end-of-life textiles is currently recycled fibre-to-fibre, and the majority is landfilled or incinerated [27,28,29], diverting the cellulosic fraction into CMC both avoids disposal emissions and displaces virgin cotton linters or wood pulp, each of which carries a substantial water and land footprint [26]. Life-cycle and eco-efficiency assessments of textile-cellulose recovery report high circularity when recycled fibre substitutes virgin input, with the principal residual burdens arising from energy and solvent consumption [30,32]. The recoverable isopropanol–water slurry used here is compatible with distillative solvent regeneration and closed-loop process design.

Beyond the observed link between feedstock cleanliness and product purity, several additional patterns emerge from the dataset. (i) Provenance affected the dispersion of product properties more than their means: the pre-consumer class had the narrowest DS distribution (SD 0.038), whereas the post-consumer class had the broadest purity distribution, with a variance 3.5 times that of the reference class. For an industrial operation, this variability determines the share of off-specification batches and the value of pre-sorting or blending. (ii) Purity correlated more strongly with log-viscosity (r = 0.57) than with DS (r = 0.38). The magnitude of the viscosity variation exceeded the change expected from polymer-concentration dilution alone: a 10-percentage-point decrease in purity corresponds to about a 13% decrease in active polymer concentration and, using a concentration exponent of 1.5–4 for semidilute Na-CMC solutions [13,44], to an estimated reduction in log η of about 0.1–0.25, whereas log η varied by more than three units within the panel. (iii) DS and log-viscosity were positively correlated (r = 0.42). (iv) Colour and substitution were decoupled: dyed fabrics gave tinted but soluble CMC with DS comparable to that of white fabrics (e.g., cornflower S4a, 0.47–0.58; black K2a, 0.44; white S3a, 0.49). A 2% (on mass of fibre) shade of a reactive dye with a molar mass of about 800 g·mol^−1^ occupies only about 0.1% of the hydroxyl groups of cellulose, consistent with the small effect on substitution. (v) The 120 min viscosity was often lower than the 90 min value, identifying reaction time as an additional viscosity-control variable. (vi) The 100% viscose garment P1a gave a DS of 0.51, within the cotton range, consistent with the dominant role of reagent stoichiometry under the slurry conditions used here (Section 3.4). Together, these observations identify sorting, etherification time and alkali reserve as practical process-control variables without changing the basic chemistry.

The comparative dataset combines feedstock, synthesis-route and reagent-ratio variation from the process-development programme, and the detailed run structure in Table S1 supports interpretation of the group-level statistics. The single-step, unpurified process produced technical-grade CMC across a broad input panel, while the literature data show that additional carboxymethylation and purification increase DS and purity [10,18,22]. The measured distributions of DS, purity and viscosity therefore provide a practical basis for feedstock sorting, route selection and grade targeting.

A mass-balance scenario based on the measured reference-product purity (75.9% at DS 0.48) illustrates the effect of retained non-cellulosic fibre on crude-product composition. Under the model in which the non-cellulosic fibre fraction w passes through the process unchanged and the by-product mass remains at the reference level, the calculated true Na-CMC content decreases from ≈76% for w = 0 to ≈74% for w = 0.03, ≈69% for w = 0.10, ≈59% for w = 0.25, ≈52% for w = 0.35 and ≈41% for a 50/50 polyester–cotton blend. The gravimetric method retains insoluble synthetic fibres in the ethanol-insoluble residue, so blended feedstocks require interpretation together with water-insoluble matter. Using about 60% active substance as the technical-grade level reported by Rahaman et al. for waste textile cotton [10], the model gives a practical non-cellulosic-fibre threshold of about 24% under the present process, or about 9% for a target purity of at least 70%. Typical cotton–elastane garments containing 2–5% elastane correspond to a calculated decrease of only 1–3 percentage points. For polyester-rich blends, alkaline hydrolysis can additionally consume NaOH and generate terephthalate species [48,49]. Separation of the water-insoluble synthetic residue and dosing of NaOH and MCA on the actual cellulose content shift the calculated product composition back toward the pure-cotton range. This mass balance therefore provides a direct basis for blend sorting and reagent dosing in mixed-textile processing.

Placed in the broader context of textile-waste valorisation, these results complement the chemical- and enzymatic-recycling routes that target regenerated fibre, glucose or platform chemicals [25,28,31]. Whereas fibre-to-fibre recycling generally requires clean, single-composition cotton and struggles with blends and dyes, the carboxymethylation route demonstrated here is comparatively tolerant of colour and moderate contamination because the product is a dissolved, purifiable polymer rather than a re-spun fibre. It therefore occupies a useful niche within the recycling hierarchy: material that is too blended, dyed or degraded for high-grade fibre regeneration can still be upgraded to a functional chemical of substantial value. This positioning integrates textile-waste-derived CMC as a complementary component of a multi-route circular-economy system for post-consumer textiles.

## 5. Conclusions

Fifty-five laboratory carboxymethylation runs on a deliberately heterogeneous panel of cotton, viscose, flax, jute and blended textile wastes—reference fabrics, pre-consumer clippings and post-consumer garments—showed that these materials can be converted into predominantly water-soluble, technical-grade Na-CMC in an isopropanol–water slurry, with DS values of 0.17–0.60 (mean 0.48). Polymer-bound carboxyl content measured in the salt-free acid form, together with product water solubility, provides chemical evidence of carboxymethylation.

No statistically significant effect of feedstock provenance on DS was detected. The equivalence analysis placed all pairwise class differences within ±0.10 DS units; at the stricter ±0.05 margin, equivalence was supported for the pre- versus post-consumer comparison, while the two comparisons involving the reference class lay at the margin of significance.

Mean purity was lower and more variable for post-consumer products (71.9 ± 7.3%) than for reference fabrics (75.9 ± 3.9%). SEM/EDS of selected cotton feedstocks revealed greater fibre damage, sulfur signals and locally elevated aluminium on the surface of the post-consumer fibres, providing a clear materials-level distinction between the feedstock classes.

Apparent 2% viscosities ranged from 2.5 to 23,100 mPa·s, and the H_2_O_2_ route consistently gave low-viscosity products (2.5–30 mPa·s). The measured range spans low- to high-viscosity CMC regimes and demonstrates process-level tunability through feedstock and route selection.

The moderate DS of all routes is consistent with the MCA charge (maximum DS of 1.15 at the baseline ratio; apparent reagent-incorporation efficiency ≈ 42%) and with competing chloroacetate hydrolysis, identifying clear process levers for reaching higher DS.

Overall, the study positions mixed textile waste as a viable secondary cellulose feedstock for technical-grade CMC, with purity and viscosity providing the main grade-selection criteria and with direct process levers available for further optimisation.

## 6. Process Development Opportunities

The results identify direct process-development opportunities. Multi-step carboxymethylation and systematically higher NaOH:MCA ratios are established routes for moving DS from the present technical-grade band (≈0.48) toward the 0.7–1.2 range used in higher-specification CMC grades [10,18,22]. In-process DS measurements can additionally map the progress of substitution alongside viscosity evolution.

Advanced characterisation can extend the structure–property map established here: size-exclusion chromatography and intrinsic-viscosity measurements of CMC, the limiting viscosity number of the parent feedstocks in cupri-ethylenediamine solution (ISO 5351), NMR analysis of substituent distribution (including HR-MAS 13C methods for non-destructive DS determination [11]), FTIR and 13C NMR structural analysis, XRD of residual crystallinity, and higher-voltage EDS/ICP-OES of purified products. Together, these measurements can connect feedstock structure, process variables and final CMC grade with greater resolution.

For blended and contaminated feedstocks, selective separation and in situ tolerance strategies—including carboxymethylation-then-dissolution and solvent-based fibre separation—offer direct routes to tighten the purity distribution of post-consumer products [23,31]. Defined polyester–cotton and elastane–cotton blends can be used to map the relationship between synthetic-fibre content and product purity [48,49].

Pilot-scale translation can combine the 12 g laboratory chemistry with a closed isopropanol–water solvent loop, quantitative solvent-regeneration balances, and life-cycle and techno-economic analysis against virgin-cellulose CMC and alternative textile-recycling routes [30,32].

Application screening can map the recovered grades to target end-uses, including rheology and stability in food/pharmaceutical formulations and electrochemical performance as aqueous anode binders in lithium-ion cells, linking the measured DS, viscosity and purity windows to market-specific performance [34,35,36].

## Figures and Tables

**Figure 1:**
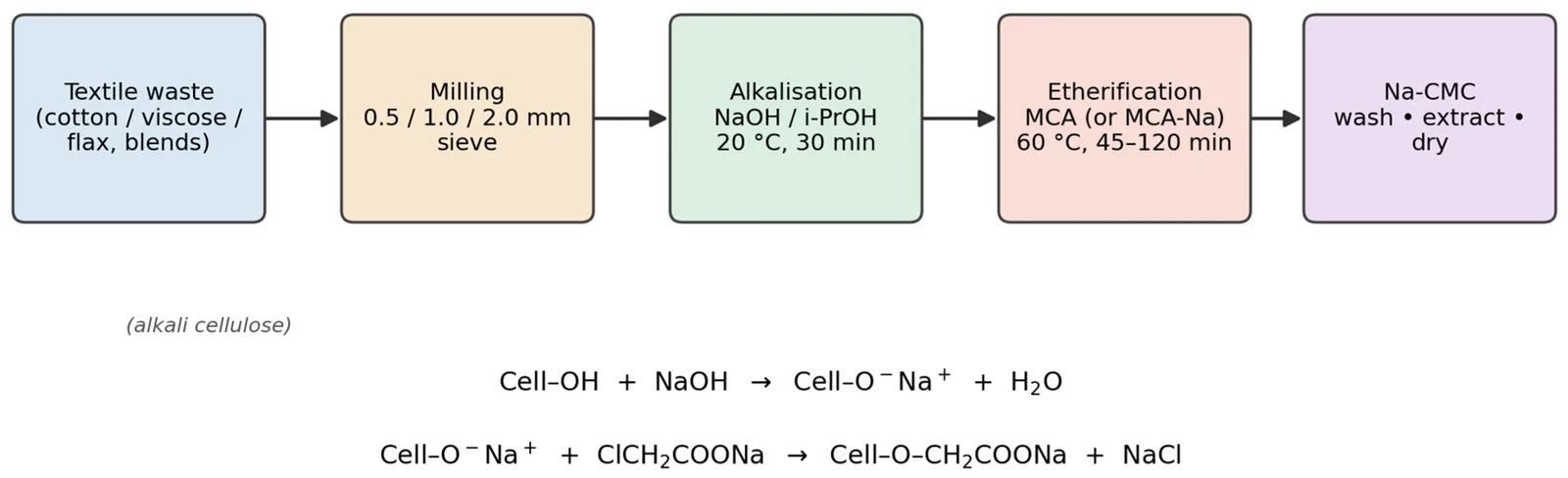
Overall workflow and reaction scheme for the conversion of cellulosic textile waste into sodium carboxymethyl cellulose (Na-CMC): mechanical shredding, alkalisation of cellulose with NaOH to form reactive alkali cellulose, and Williamson etherification with monochloroacetic acid (or its sodium salt) in an isopropanol–water slurry. Coloured boxes indicate successive process stages; arrows indicate process flow.

**Figure 2:**
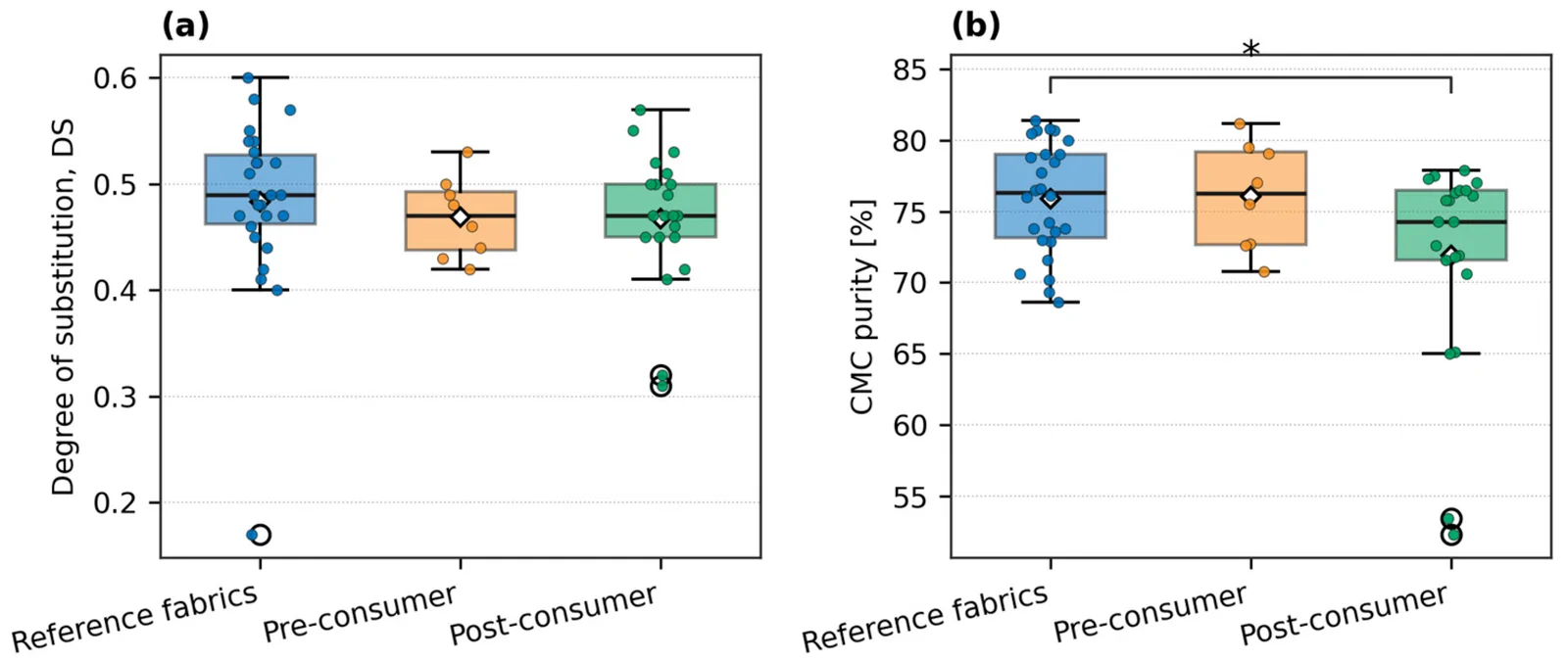
Distribution of (**a**) degree of substitution (DS) and (**b**) carboxymethyl cellulose (CMC) purity by feedstock class. Blue, orange and green denote reference, pre-consumer and post-consumer feedstocks, respectively. Boxes show interquartile range and median; white diamonds mark the mean; points are individual syntheses. DS does not differ significantly among classes, whereas post-consumer (P) purity is significantly lower than that of reference fabrics (S) (Tukey honestly significant difference (HSD), * *p* < 0.05); this difference was not significant when the unequal variances were taken into account (Welch’s analysis of variance (ANOVA), *p* = 0.093; Games–Howell, *p* = 0.077; Section 3.2).

**Figure 3:**
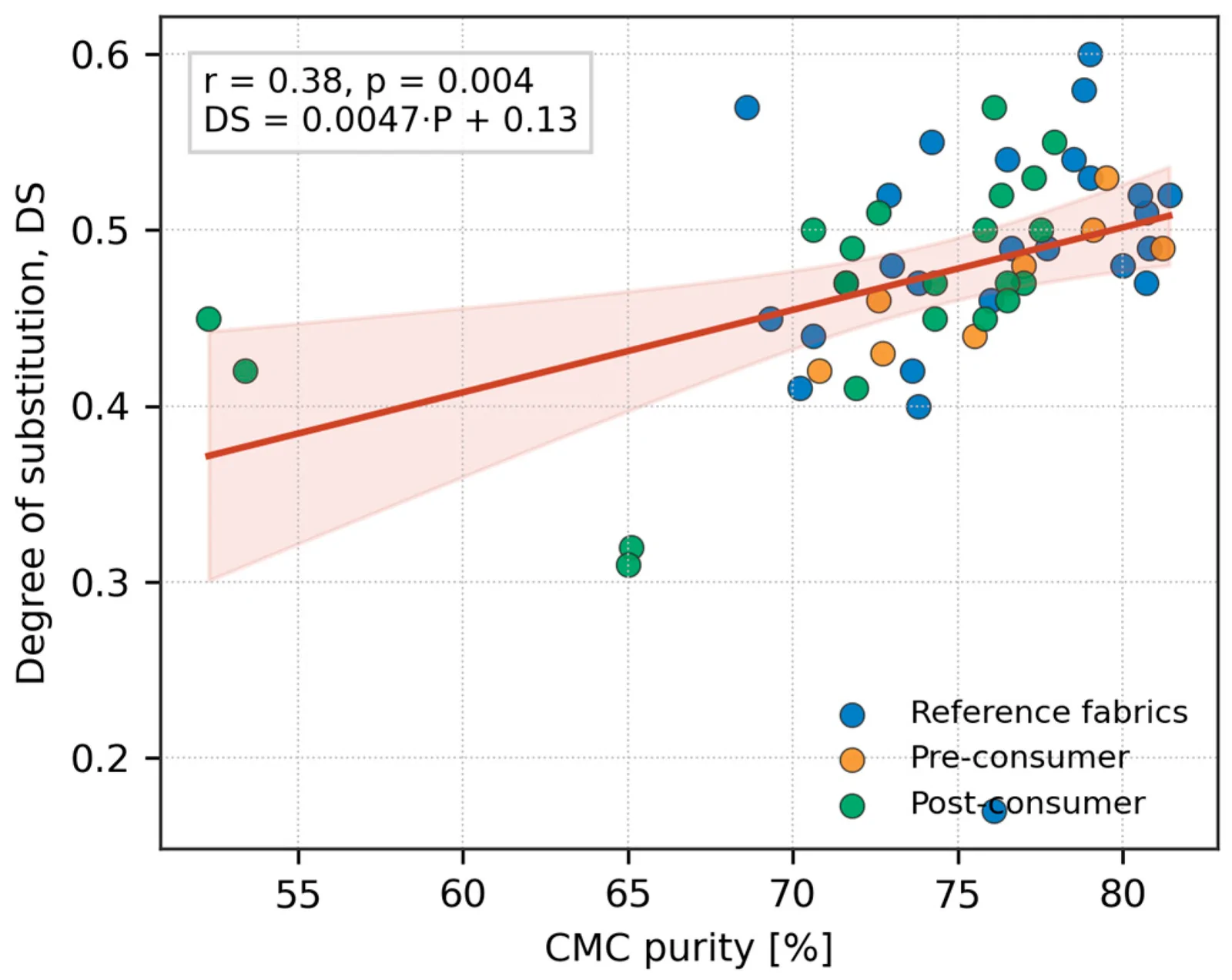
Degree of substitution (DS) versus carboxymethyl cellulose (CMC) purity for all 55 syntheses, coloured with blue, orange and green points denoting reference, pre-consumer and post-consumer feedstocks, respectively, with the ordinary least-squares regression line (red) and its 95% confidence band. The positive, significant relationship (r = 0.38, *p* = 0.004) is consistent with more effective substitution in cleaner cellulose but does not establish a causal relationship.

**Figure 4:**
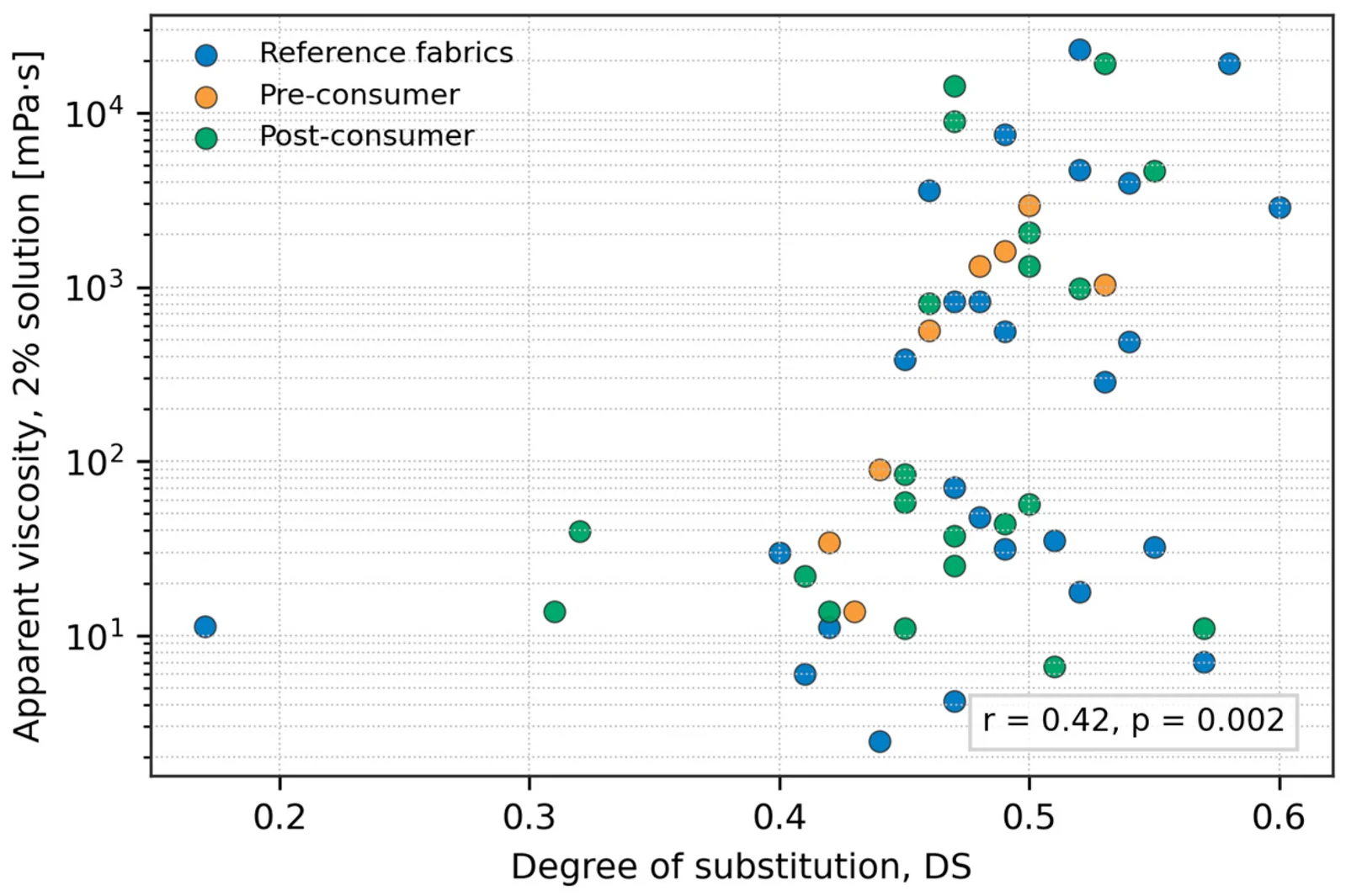
Apparent viscosity of 2% carboxymethyl cellulose (CMC) solutions (logarithmic axis) versus degree of substitution (DS), coloured with blue, orange and green circles denoting reference, pre-consumer and post-consumer feedstocks, respectively. Viscosity spans four orders of magnitude independently of provenance and correlates positively with DS (r = 0.42, *p* = 0.002).

**Figure 5:**
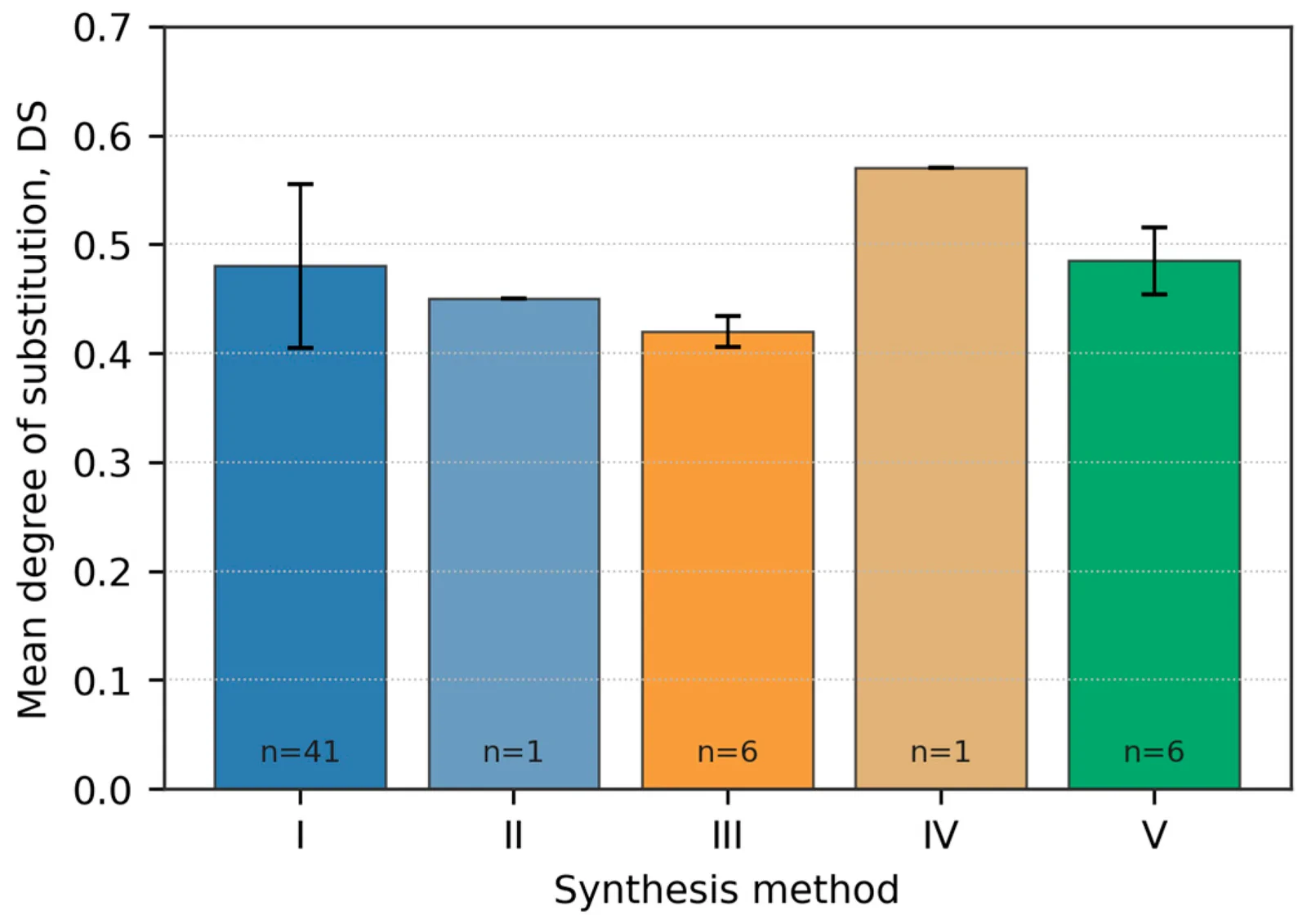
Mean degree of substitution (DS) by synthesis route (I—baseline; II—stepwise reagents; III—hydrogen peroxide (H_2_O_2_) bleaching/degrading; IV—dichloroacetic acid (DCA) cross-linking; V—sodium monochloroacetate (MCA-Na)). Bar colours distinguish Routes I–V; error bars are standard deviations; *n* indicates the number of syntheses. Route had no significant effect on DS (analysis of variance (ANOVA) on I/III/V, *p* = 0.13).

**Figure 6:**
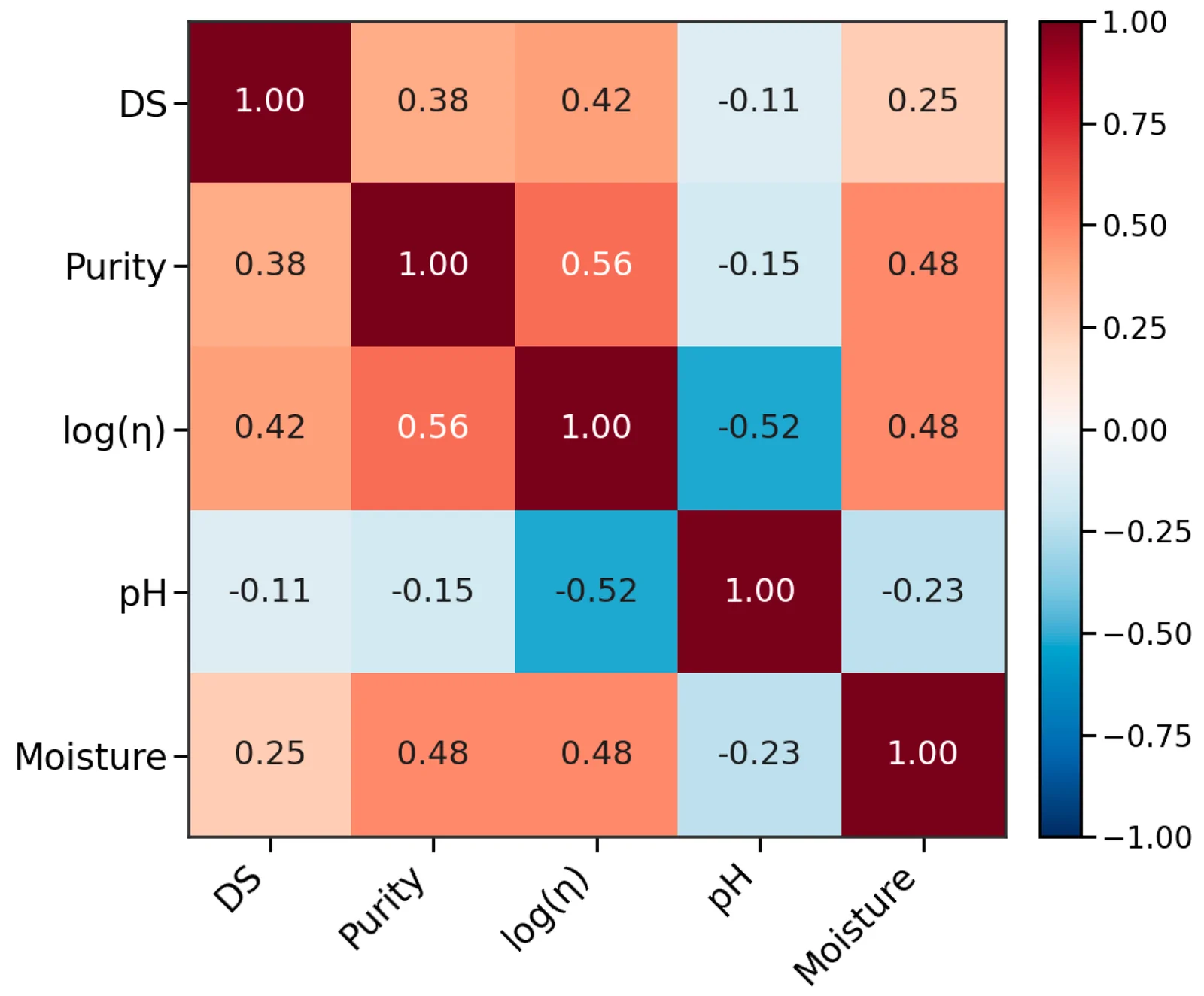
Pearson correlation matrix for the measured product properties (η = apparent viscosity, shown as log_10_). Degree of substitution (DS)–purity, DS–log(η) and purity–log(η) are all positive and statistically significant. Red denotes positive and blue negative correlations; colour intensity reflects the magnitude of Pearson’s r.

**Figure 7:**
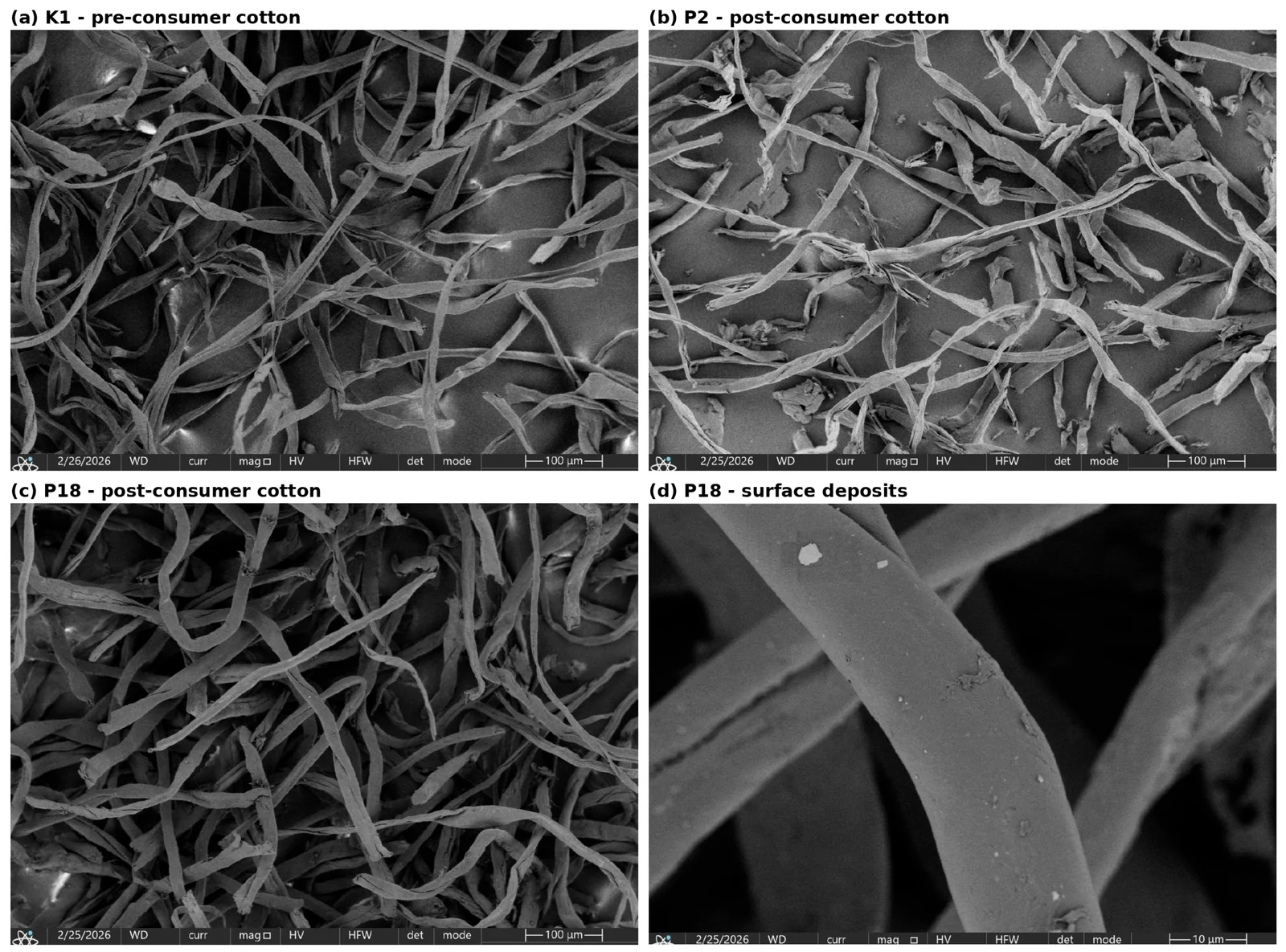
Representative scanning electron microscopy (SEM) micrographs of shredded cotton feedstocks acquired at 2 kV. (**a**) Pre-consumer cotton K1: smooth, clean, ribbon-like fibres with intact ends. (**b**) Post-consumer cotton P2 and (**c**) post-consumer cotton P18: frayed, split and roughened fibres with adhering debris. (**d**) Higher-magnification view of a P18 fibre showing bright particulate surface deposits. Scale bars and acquisition parameters are printed on each panel.

**Table 1:** Representative cellulosic feedstocks used for sodium carboxymethyl cellulose (CMC) synthesis, grouped by provenance class (S = reference fabrics; K = pre-consumer waste; P = post-consumer waste). Fibre compositions are as declared by the supplier or on the garment care label.

Class	Code	Composition	Colour	Notes/Supplier
S	S1a	Raw flax—100%	natural	VIVE Innovation
S	S2a	Raw jute—100%	natural	VIVE Innovation
S	S3a	Cotton—100%	white	150 g·m^−2^, Fashion Colours
S	S4a	Cotton—100%	cornflower	125 g·m^−2^, Fashion Colours
S	S10a	Viscose 68%/Cotton 32%	navy floral	200 g·m^−2^, TEONA
S	S12a,b	Cotton 97%/Elastane 3%	white	250 g·m^−2^, TEONA
S	S14a,c	Flax—100%	white floral	140 g·m^−2^, TEONA
S	S15b	Arbocel B400 (MCC)	white	J. Rettenmaier & Söhne
K	K1a	Cotton—100%	white	Hosokawa Alpine milled, Radom
K	K2a	Cotton—100%	black	Hosokawa Alpine milled, Radom
K	K3a,c	Cotton—100%	white	Sewing-room clippings, Radom
P	P1a	Viscose—100%	white/black	Dress, VIVE
P	P2a,b	Cotton—100%	white	T-shirt wiper, Herbort milled, VIVE
P	P4c	Cotton 98%/Elastane 2%	beige	Trousers (Jack&Jones), VIVE
P	P11a,b	Cotton—100%	white	T-shirt (Boss), VIVE
P	P13b,c	Cotton 75%/Flax 25%	salmon	Blouse, VIVE
P	P18a–c	Cotton—100% (0.355/0.5/0.8 mm)	white	Bedding+flannel, Pallmann milled, VIVE

**Table 2:** Representative synthesis conditions and product properties (η is the 2% apparent viscosity of the final 120-min product, measured at 50 min^−1^ after Soxhlet extraction). The full dataset (*N* = 55) is summarised statistically in Table 3 and given in full in Supplementary Table S1. DS denotes degree of substitution.

Run	Class	Feedstock	Method	DS	η 2% at 50 min^−1^ [mPa·s]	Purity [%]	pH	Moist. [%]
I.1	S	Raw flax (S1a)	I	0.57	7.1	68.6	9.0	6.2
I.3	S	Cotton white (S3a)	I	0.49	31.4	77.7	9.6	8.8
I.4	S	Cotton (S4a)	I	0.47	71.3	71.6	9.2	9.6
I.17	S	Cotton/elastane (S12a)	I	0.52	4681.7	80.5	6.4	8.2
I.21	S	Flax (S14c)	I	0.52	23,102.1	81.4	7.1	11.4
I.27	S	Cotton (S4a)	I	0.58	19,279.0	78.8	10.3	4.4
I.8	S	Cotton (S6a)	III	0.44	2.5	70.6	8.0	2.6
I.12	S	Cotton (S4a)	V	0.54	485.3	76.5	7.5	8.9
II.2	K	Cotton Alpine (K2a)	I	0.44	89.5	75.5	10.5	1.4
II.6	K	Cotton Alpine (K1a)	I	0.49	1615.0	81.2	7.3	5.3
II.9	K	Cotton (K3b)	I	0.53	1033.0	79.5	7.0	9.7
III.1	P	Viscose (P1a)	I	0.51	6.6	72.6	9.5	6.9
III.4	P	Cotton (P3a)	I	0.42	13.7	53.4	8.5	3.0
III.11	P	Cotton (P7c)	I	0.55	4654.5	77.9	5.7	5.0
III.16	P	Cotton (P11a)	I	0.47	14,251.5	76.5	6.6	8.3
III.13	P	Cotton (P8c)	I	0.32	39.5	65.1	7.1	2.5
III.20	P	Cotton (P7c)	V	0.50	2059.2	75.8	8.0	9.4

**Table 3:** Descriptive statistics (mean ± standard deviation (SD)) by feedstock class and one-way analysis of variance (ANOVA) results. Superscript letters denote Tukey honestly significant difference (HSD) groupings (α = 0.05); shared letters indicate no significant difference. Asterisk marks a significant class effect (*p* < 0.05). Welch’s heteroscedasticity-robust analysis of variance (ANOVA): degree of substitution (DS), F(2,27.4) = 0.29, *p* = 0.75; purity, F(2,20.4) = 2.67, *p* = 0.093; log_10_(η), F(2,23.5) = 0.23, *p* = 0.79 (Section 2.6).

Property	S (*n* = 26)	K (*n* = 8)	P (*n* = 21)	ANOVA (F, p)
DS	0.483 ± 0.082 ^a^	0.469 ± 0.038 ^a^	0.468 ± 0.064 ^a^	F = 0.33, *p* = 0.72
Purity [%]	75.9 ± 3.9 ^a^	76.1 ± 3.8 ^ab^	71.9 ± 7.3 ^b^	F = 3.59, *p* = 0.035 *
log_10_(η 2%)	2.11 ± 1.14 ^a^	2.34 ± 0.79 ^a^	2.12 ± 1.20 ^a^	F = 0.22, *p* = 0.81
pH	8.48 ± 1.30	7.88 ± 1.69	8.04 ± 1.35	–
Moisture [%]	6.74 ± 3.19	5.66 ± 3.11	5.88 ± 2.09	–

**Table 4:** Surface energy-dispersive X-ray spectroscopy (EDS) microanalysis (atomic %) of representative pre-consumer (K1) and post-consumer (P2, P18) cotton feedstocks, acquired at 2 kV. Values are per-sample averages; B, N and light elements are semi-quantitative at this accelerating voltage. Elevated S (P2b) and Al (P18b) indicate non-cellulosic species on the surface of the post-consumer fibres; at 2 kV the analysis probes only the near-surface region of the raw feedstock and does not describe the carboxymethyl cellulose (CMC) products (see text).

Sample	B	N	Al	C	O	S
*K1a*	0.6	3.0	0.2	51.7	44.4	0.0
*K2a*	0.7	3.6	0.1	57.2	38.4	0.0
*P2a*	0.7	3.3	0.1	56.7	39.2	0.0
*P2b*	0.7	2.9	0.2	50.8	45.2	0.3
*P18a*	0.7	3.6	0.2	55.3	40.0	0.1
*P18b*	0.6	3.5	0.6	49.9	45.3	0.1
*P18c*	0.7	3.3	0.1	54.3	41.6	0.0

## Data Availability

The original contributions presented in this study are included in the article/Supplementary Material. Further inquiries can be directed to the corresponding author.

## Supplementary Materials

The following supporting information can be downloaded at: https://www.mdpi.com/article/10.3390/ma19194101/s1, Table S1: Complete synthesis conditions and product properties for the 55-run comparative dataset.
